# CD147 regulates extrinsic apoptosis in spermatocytes by modulating NFκB signaling pathways

**DOI:** 10.18632/oncotarget.13624

**Published:** 2016-11-25

**Authors:** Chaoqun Wang, Kin Lam Fok, Zhiming Cai, Hao Chen, Hsiao Chang Chan

**Affiliations:** ^1^ Epithelial Cell Biology Research Center, Key Laboratory for Regenerative Medicine of The Ministry of Education of China, School of Biomedical Sciences, Faculty of Medicine, The Chinese University of Hong Kong, Shatin, Hong Kong; ^2^ Department of Gynecology, The Second People's Hospital of Shenzhen, Shenzhen, PR China; ^3^ Sichuan University – The Chinese University of Hong Kong Joint Laboratory for Reproductive Medicine, West China Second University Hospital, Sichuan University, Chengdu, China

**Keywords:** CD147, non-canonical NFκB, apoptosis, spermatocytes, spermatogenesis

## Abstract

CD147 null mutant male mice are infertile with arrested spermatogenesis and increased apoptotic germ cells. Our previous studies have shown that CD147 prevents apoptosis in mouse spermatocytes but not spermatogonia. However, the underlying mechanism remains elusive. In the present study, we aim to determine the CD147-regulated apoptotic pathway in mouse spermatocytes. Our results showed that immunodepletion of CD147 triggered apoptosis through extrinsic apoptotic pathway in mouse testis and spermatocyte cell line (GC-2 cells), accompanied by activation of non-canonical NFκB signaling and suppression of canonical NFκB signaling. Furthermore, CD147 was found to interact with TRAF2, a factor known to regulate NFκB and extrinsic apoptotic signaling, and interfering CD147 led to the decrease of TRAF2. Consistently, depletion of CD147 by CRISPR/Cas9 technique in GC-2 cells down-regulated TRAF2 and resulted in cell death with suppressed canonical NFκB and activated non-canonical NFκB signaling. On the contrary, interfering of CD147 had no effect on NFκB signaling pathways as well as TRAF2 protein level in mouse spermatogonia cell line (GC-1 cells). Taken together, these results suggested that CD147 plays a key role in reducing extrinsic apoptosis in spermatocytes, but not spermatogonia, through modulating NFκB signaling pathway.

## INTRODUCTION

Spermatogenesis is a complicated process that involves a series of cellular events, stepping from spermatogonia, spermatocytes, spermatids to spermatozoa [1, 2]. Interestingly, a large number of genes expressed in developing germ cells are eventually shown to be oncogenes, which induce tumorigenesis. Those genes are also known as cancer/testis (CT) antigens [3]. It has been proposed that reactivation of gametogenic genes is one of the driving forces of tumorigenesis [4], and that the processes of cancer development and spermatogenesis shares critical similarities [5].

CD147, a highly glycosylated protein, is abundantly expressed in the reproductive tracts and most of cancers, including breast cancer, liver cancer, gastric cancer, lung cancer, melanoma, ovarian cancer and testicular cancer [6, 7]. It has been well documented to promote tumorigenesis and metastasis through enhancing cell migration and invasion and conferring resistance to chemotherapeutic drugs [8, 9]. The role of CD147 in cancer migration has been associated with: the regulation of matrix metalloproteinases (MMPs), the essential enzymes for extracellular matrix (ECM) degradation [7, 10]; the rearrangement of cytoskeleton that mediated mesenchymal movement; and the activation of other molecular pathway including uPA, intergrin-FAK-PI3K and TGF-beta [11–13]. On the other hand, reactivation of CD147 in tumors has been implicated in tumor growth through its capability to enhance the survival of cancer cells [9, 14]. Thus, CD147 is considered as an essential tumor-specific marker in many cancers [15]. In adult mouse testis, CD147 is expressed on the cell surface of spermatogonia and spermatocytes, and gradually increases during the meiotic prophase [3, 16]. Knockout of CD147 leads to azoospermic phenotype, indicating that this gene is essential for spermatogenesis [17]. Spermatogenesis was arrested at the metaphase of the first meiotic division in CD147 null mutant mice. The lumens of the male reproductive system were filled with round degenerated cells, some of which were undergoing apoptosis [18].

Apoptosis is designed to remove the abnormal and excess germ cells, defects of which can cause azoospermia or neoplasia in human [19, 20]. It has been shown that about 75% of them were eliminated at different stages [21]. Both intrinsic and extrinsic apoptotic pathways are involved in the regulatory network of germ cell apoptosis. The intrinsic pathway is mainly mediated by Bcl-2 family members, including Bax and Bcl-2; the extrinsic pathway is initiated by the binding of death ligand and death receptor and formation of death-inducing signaling complex (DISC), resulting in the cleavage of procaspase 8 and ultimately activation of caspase 3 [22].

NFκB signaling is well-known to regulate apoptosis in cancer cells. Two NFκB signaling pathways have been discovered, the canonical pathway (predominantly mediated by p50/p65 dimer) and the non-canonical pathway (mediated by p52/RelB dimer) [23]. Activation of the canonical pathway relies on the degradation of inhibitor of NFκB (IκBs), leading to nuclear translocation of p50/p65 dimer [24]. Non-canonical pathway is activated by NFκB-inducing kinase (NIK), generating p52 from p100, followed by p52/RelB nuclear translocation [25, 26]. Notably, TRAF2 positively regulates the canonical pathway by inducing IκBs degradation and negatively regulates the non-canonical pathway through promoting NIK degradation [27, 28]. In the testis, canonical NFκB factors, p50 and p65, are transiently expressed in the nuclei of germ cells with peak levels found in the pachytene spermatocytes during spermatogenesis, indicating that this might be required for gene induction during this stage [29]. Further study has shown that aberrant activation of canonical NFκB signaling, induced by knockout of its negative regulator CYLD, contributed to the suppression of apoptosis in spermatogonia and spermatocytes at early stages of spermatogenesis [30]. Our previous studies have also shown that interference of CD147 by its antibody reduced the number of spermatogenic cells, which was associated with increased apoptosis. Interestingly, cell apoptosis mainly occurred in spermatocytes but not spermatogonia [31]. We have further confirmed that CD147 is involved in regulating spermatocytes apoptosis in a p53, Bax and Bcl-2 independent manner [31]. However, how CD147 is involved in the signaling network regulating germ cell apoptosis remains unknown. Interestingly, it has been reported that CD147 could bind to TRAF2 for enhancing cancer metastasis [32]. Furthermore, TRAF2 is also involved in the regulation of extrinsic apoptosis and NFκB signaling pathways [28, 33]. Together, these studies prompt us to hypothesize that CD147 regulates spermatocytes apoptosis through the TRAF2-regulated extrinsic apoptotic pathway and NFκB signaling pathways. We undertook the present study to examine this hypothesis and to determine the molecular mechanism underlying different apoptotic roles of CD147 in spermatocytes and spermatogonia.

## RESULTS

### Immunodepletion of CD147 activates the extrinsic apoptotic pathway in spermatocytes by decreasing TRAF2

**Figure 1:**
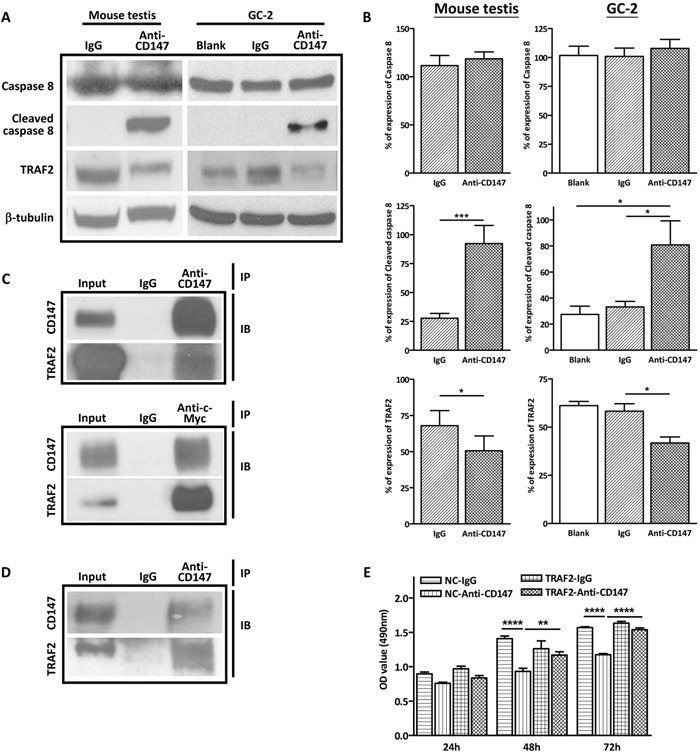
Activation of the extrinsic apoptosis in anti-CD147 treated GC-2 cells and mouse testis **A**. Representative images of western blot analysis of the extrinsic apoptotic factors TRAF2, procaspase 8 and cleaved caspase 8 in CD147-immunodepleted testis of SCID mouse (left panel) and anti-CD147 treated GC-2 cells (right panel). The GC-2 cells were treated with 10 μg/mL anti-CD147 for 48 h. The testis was injected with 10μl mouse anti-CD147 mAb (40 μg/mL) and the total protein of testes was harvested after nine days. β-tubulin was used as the loading control. **B**. The corresponding statistical analysis (*, *p*<0.05 *vs* IgG control), the experiments were repeated 3 times. Values represent the mean±SEM. **C**. Co-immunopreciaptation (IP) of TRAF2 and CD147 in HEK293 cells. Myc-tagged TRAF2 was transfected into HEK293 cells and cell lysate was extracted with IP lysis buffer after 48 h transfection. TRAF2 or CD147 was pull down by indicated antibodies and the interaction was detected by immunoblot (IB) for CD147 and myc-tagged TRAF2. **D**. Co-immunoprecipitation (IP) of TRAF2 and CD147 in GC-2 cells. Endogenous TRAF2 was pulled down by anti-CD147 antibody and the interaction was determined by immunoblotting (IB) for CD147 and TRAF2. **E**. Overexpression of TRAF2 ameliorates the decrease in viability of CD147-depleted cells. GC-2 cells were transfected with TRAF2 overexpressing plasmid or vector control and treated with anti-CD147 antibody (10 ug/ml) or normal IgG. Summary of MTS assay (OD490 nm) at indicated time points is shown. (****, *p*<0.001 *vs* IgG control, **, *p*<0.01 *vs* IgG control).

Immunodepletion of CD147 was used in several studies for blocking functional CD147 [34, 35]. The CD147 neutralizing antibody used in the present study recognizes the extracellular domain of CD147 and blocks its biological functions [31, 34]. Our previous study has shown that CD147 regulates spermatocytes apoptosis independent of p53 as well as the intrinsic apoptotic factors Bcl-2 and Bax [3]. Thus, we set out to explore the possibility that CD147 regulates spermatocytes apoptosis through the extrinsic apoptotic pathway. The extrinsic apoptotic marker cleaved caspase 8 was examined in the CD147-immunodepleted mouse testis and GC-2 cells by western blot analysis. As shown in Figure 1A and 1B, increased level of cleaved caspase 8 was observed in both anti-CD147 antibody-treated GC-2 cells and anti-CD147 antibody- injected mouse testis, indicating the activation of the extrinsic apoptosis. Previous studies have shown that CD147 could bind to the extrinsic apoptotic inhibitor TRAF2 [32, 33]. We first validated the interaction between CD147 and TRAF2 by co-immunoprecipitation in HEK293 cells overexpressing myc-tagged TRAF2. Our results showed that myc-tagged TRAF2 was pulled down by CD147 antibodies, but not the IgG control. Similarly, CD147 was pulled down by c-myc antibody, which recognizes the myc-tagged TRAF2 (Figure 1C). Furthermore, TRAF2 was also pulled down by CD147 antibodies in GC-2 cells (Figure 1D), suggesting endogenous interaction of CD147 and TRAF2 in spermatocytes. Since CD147 interacts with TRAF2, we speculated that inference with CD147 could affect the expression of TRAF2, which in turn leads to extrinsic apoptosis. Indeed, both western blot and real-time PCR results showed that anti-CD147 antibody treatment reduced the level of TRAF2 in both GC-2 cells and mouse testis (Figure 1A and 1B, Supplementary Figure S1), suggesting that the activation of the extrinsic apoptotic pathway might be attributed to the decreased TRAF2. In corroboration to this idea, MTS assay showed that anti-CD147 antibody treatment significantly decreased the number of viable GC-2 cells, while overexpression of TRAF2 prevented the loss of cell viability induced by anti-CD147 antibody treatment in a time-dependent manner (Figure 1E). Taken together, these results suggest that CD147 regulates the extrinsic apoptosis in spermatocytes by modulating the level of TRAF2.

### Interference with CD147 function suppresses canonical NFκB signaling in spermatocytes

**Figure 2:**
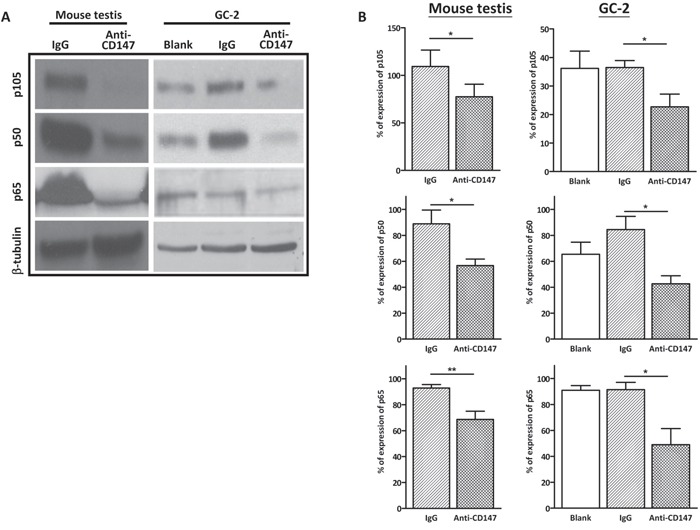
Immunodepletion of CD147 suppresses the canonical NFκB signaling **A**. Representative images of western blot analysis of the canonical NFκB factors p105, p50 and p65 in CD147-immunodepleted testis and anti-CD147 treated GC-2 cells. The GC-2 cells were treated with 10 μg/mL anti-CD147 for 48 h. The testis was injected with 10μl mouse anti-CD147 mAb (40 μg/mL) and the total protein of testes was harvested after nine days. β-tubulin was used as the loading control. **B**. The corresponding statistical analysis (*, *p*<0.05 *vs* IgG control), the experiments were repeated 3 times. Values represent the mean±SEM.

TRAF2 is known to stimulate canonical NFκB signaling, which is known to suppress apoptosis. Since depletion of CD147 reduces the level of TRAF2, we evaluated the alteration of canonical NFκB factors in the CD147 immunodepleted GC-2 cells and mouse testis. Consistent with the activation of cleaved caspase 3 in CD147 immunodepleted germ cells [31], the expression of canonical NFκB factors p105, p50 and p65 was decreased both in the CD147 immunodepleted GC-2 cells and mouse testis, compared with the IgG groups (Figure 2). These results suggest that interference of CD147 suppresses canonical NFκB signaling in spermatocytes.

### Interference with CD147 function activates non-canonical NFκB signaling in spermatocytes

**Figure 3:**
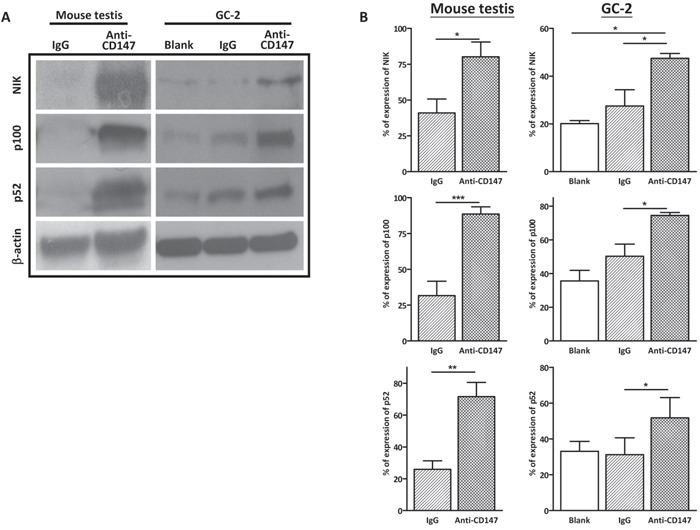
Immunodepletion of CD147 activates the noncanonical NFκB signaling **A**. Representative images of western blot analysis of the noncanonical NFκB factors NIK, p100 and p52 in CD147-immunodepleted testis and anti-CD147 treated GC-2 cells. The testis was injected with 10μl mouse anti-CD147 mAb (40 μg/mL) and the total protein of testes was harvested after nine days. The GC-2 cells were treated with 10 μg/mL anti-CD147 for 48 h. β-tubulin was used as the loading control. **B**. The corresponding statistical analysis (*, *p*<0.05 *vs* IgG control), the experiments were repeated 3 times. Values represent the mean±SEM.

Apart from the canonical NFκB pathway, TRAF2 also negatively regulates the non-canonical NFκB signaling, which has been implicated in the activation of the extrinsic apoptosis, by inducing the degradation of NIK [27, 36, 37]. NIK activates non-canonical NFκB signaling by promoting the processing of p100 to p52, followed by p52/RelB nuclear translocation [25, 26]. To examine the activation of non-canonical NFκB by immunudepletion of CD147, the protein levels of non-canonical NFκB factors, including NIK, p100 and p52, were examined by western blot in the CD147-immunodepleted GC-2 cells and mouse testis. The results showed that the protein level of NIK increased dramatically in both CD147-immunodepleted GC-2 cells and mouse testis (Figure 3A and 3B), followed by activation of non-canonical NFκB signaling with elevated p100 and p52, compared with IgG controls. Taken together, these results suggest that interference of CD147 with its antibody stimulates apoptosis via non-canonical NFκB signaling in spermatocytes.

### Knockout of CD147 with CRISPR/Cas9 mimics the effect of anti-CD147 antibody in GC-2 cells

**Figure 4:**
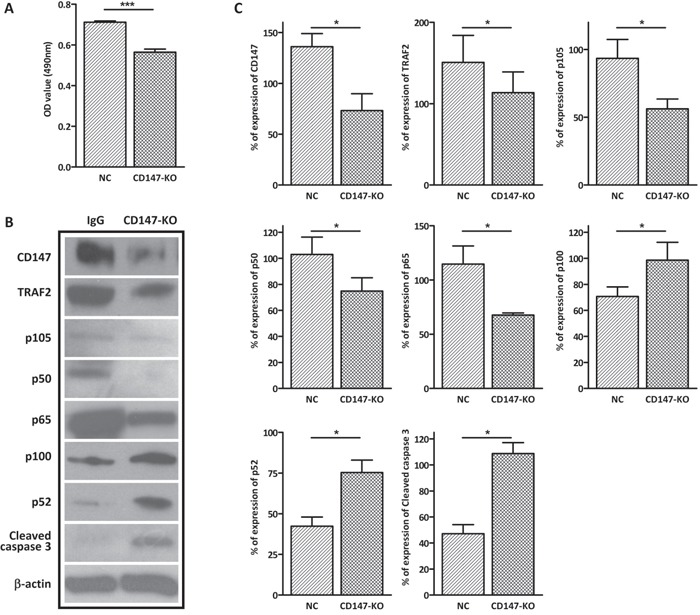
Knockout of CD147 by CRISPR/Cas9 resembles the effect of CD147 immunodepletion **A**. MTS cell viability assay showing the effect of CRISPR/Cas9-mediated CD147 knockout on GC-2 cell viability. GC-2-Cas9 cells transfected with gRNA1/2 displayed reduced cell viability after 48 h, compared with the negative control (NC). **B**. Representative images of western blot analysis of the canonical NFsB factors (p105, p50 and p65), the noncanonical NFκB factors (p100 and p52) and apoptosis markers (cleaved caspase 3) in gRNA1/2 treasfected GC-2-Cas9 cells. β-actin was used as the loading control. **C**. The corresponding statistical analysis for the western blot (*, *p*<0.05 *vs* IgG control), the experiments were repeated 3 times. Values represent the mean±SEM.

To confirm the effect of the anti-CD147 antibody on extrinsic apoptosis and NFκB signaling, we knockout CD147 in GC-2 cells by CRISPR/Cas9 technique and examined the effect of CD147 knockout on GC-2 cells. We designed two short guide RNAs (sgRNA) that target the 5’ proximal region of exon 2 and the 3’ proximal region of exon 4 of mouse *CD147* gene. After non-homologous end joining, exon 2 – 4 of *CD147* gene will be removed which leads to the frameshift mutation of CD147 gene (Supplementary Figure S2A). Knockout of CD147 was confirmed by genomic PCR, which showed the presence of the recombined allele in GC-2 cells transduced with Cas9 nuclease and the two sgRNAs (Supplementary Figure S2B). The MTS cell viability assay showed that knockout of CD147 significantly decreased cell viability in GC-2 cells (Figure 4A). Consistent with the results in CD147-immunodepletion experiments, western blot analysis showed that knockout of CD147 induced the down-regulation of TRAF2, accompanied by decreased p105, p50 and p65, indicating that the loss of CD147 suppressed canonical NFκB signaling in GC-2 cells (Figure 4B and 4C). Simultaneously, non-canonical NFκB factor p100 and p52 were up-regulated in the CD147 knockout GC-2 cells, compared with the negative control (Figure 4B and 4C). In addition, cleaved caspase 3 was activated in the CD147 knockout GC-2 cells, indicating activation of apoptosis (Figure 4B and 4C). Taken together, these results showed that knockout of CD147 had the same the effect as CD147 immunodepletion, suggesting that the anti-CD147 antibody treatment is effective and specific in blocking the function of CD147.

### Interference with CD147 function has no effect on TRAF2 in spermatogonia

**Figure 5:**
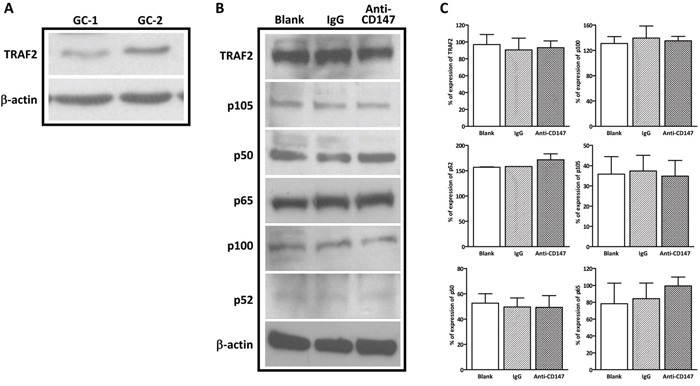
Immunodepletion of CD147 has no effect on the NFkB signaling in spermatogonia **A**. Endogenous expression of TRAF2 in GC-1 and GC-2 cells.β-actin was used as the loading control. **B**. The expression of TRAF2, NFκB factors NIK, p100, p52, p105, p50, and p65 showed no significant change in anti-CD147 antibody (10 ug/ml) treated GC-1 cells compared to IgG control. **C**. The corresponding statistical analysis, the experiments were repeated 3 times. Values represent the mean±SEM.

Our previous study has shown that anti-CD147 antibody did not induce apoptosis in mouse spermatogonia [31]. Thus, we reasoned that the expression of TRAF2 in GC-1 cells might be different from GC-2 cells. Indeed, the expression of TRAF2 in GC-1 cells was considerably lower than that in GC-2 cells (Figure 5A). This result suggests CD147 is dispensable in maintaining the protein level of TRAF2 and its downstream canonical and non-canonical NFκB factors would not be altered by immunodepletion of CD147 in mouse spermatogonia GC-1 cells. Indeed, there was no significant difference in TRAF2 protein levels between the anti-CD147 treatment group and the IgG control, as well as blank control (Figure 5B and 5C). We further examined the alteration of canonical NFκB factors p105, p50 and p65 and non-canonical NFκB factors p100 and p52 in anti-CD147 antibody treated GC-1 cells. Similarly, there was no significant alteration between anti-CD147 treatment and IgG and blank controls (Figure 5B and 5C). Since depleting CD147 did not induce apoptosis in GC-1 cells, the expression of cleaved caspase 3 was not detected (data not shown). Collectively, these results indicate that CD147 deprivation modulates NFκB pathway in spermatocyte but not spermatogonia.

## DISCUSSION

Accumulating evidences have demonstrated that CD147 acts as a pro-survival factor in many cell types including cancer cells, endometrial cells and spermatocytes by inhibiting apoptosis [7, 38, 39]. Interestingly, CD147 exerts its anti-apoptotic function through diverse signaling pathways in different cell types. CD147 has been shown to contribute to the chemoresistance of anti-cancer drug cisplatin in head and neck squamous cell carcinoma cells, which might be mediated through its interaction with urokinase plasminogen activator receptor [40]. In hepatocellular carcinoma, it is reported that CD147 activates the unfolded protein response to inhibit apoptosis and increase chemoresistance by enhancing the transcription of Bip [9]. Blocking of CD147 has also been reported to induce cell death in colon cancer cells and melanoma cells *via* interacting with monocarboxylate transporter (MCT-1), which is required for lactate transport and glycolytic energy metabolism [34]. In the progression of endometriosis, it has been shown that CD147 enhances cell viability of human endometrial cells by up-regulating Bcl-2 through extracellular signal-regulated kinases signaling [38]. The ability of CD147 to regulate diverse signaling pathways involved in apoptosis could be attributed to its ability to interact with different proteins.

Our previous studies have shown that CD147 is critical for the survival of spermatocytes [31] apart from its regulating the migration of germ cells [3]. Bi *et al*. have demonstrated that loss of CD147 led to germ cells apoptosis accompanying by reduced N-acetylglucosamine (GlcNAc) terminated N-glycans that was considered to participate in germ cell-Sertoli cell adhesion [41], suggesting that CD147-regulated germ cell apoptosis may be attributed to the impairment of the adhesions between germ cells and Sertoli cells.

In the present study, we have demonstrated that interfering with CD147 function results in the activation of extrinsic apoptosis signaling both in CD147-immunodelepted mouse testis and GC-2 cells. Also, we found that CD147 regulates the extrinsic apoptosis specifically in spermatocytes but not spermatogonia, as evidenced by a much lower expression of TRAF2 in GC-1 cells compared to GC-2 cells (Figure 5A), suggesting that CD147-TRAF2 interaction is dispensable for spermatogonia cell survival. The anti-apoptotic function of CD147 is mediated by its interacting partner TRAF2 in spermatocytes, which activates the canonical NFκB signaling while suppresses the noncanonical NFκB signaling. These findings have provided a possible mechanism underlying the regulatory effect of CD147 on spermatocyte apoptosis. Questions as to how CD147 decreases TRAF2 and whether the regulation involves the adhesions between germ cells and Sertoli cells await further investigation.

TRAF2 is known to activate the canonical while suppresses the non-canonical NFκB signaling pathways [28]. Canonical NFκB signaling, as a pro-survival factor, promotes the expression of anti-apoptotic factors, including Bcl-2, c-FLIP and cellular inhibitor of apoptosis (c-IAP) [42, 43]. During spermatogenesis, canonical NFκB signaling is activated in spermatocytes and remains active in the following steps of germ cell differentiation and maturation, suggesting its potential involvement in regulating spermatogenesis [29, 44]. The deubiquitinating enzyme CYLD negatively regulates canonical NFκB signaling, and loss of CYLD in testicular cells leads to constitutive activation of canonical NFκB signaling and aberrant expression of anti-apoptotic factors, including Bcl-2, Bcl-XL, c-IAP1 and c-IAP2, in different stages of testicular cells [30]. While our previous study has shown that depletion of functional CD147 induces apoptosis in spermatocytes but not spermatogonia [31], the current study has further demonstrated that CD147 deprivation suppresses canonical NFκB signaling specifically in spermatocytes. Interestingly, despite the suppression of canonical NFκB signaling, the expression of Bcl-2 is maintained in CD147-immunodepleted spermatocytes [31]. These results suggest that CD147-regulated canonical NFκB signaling is dispensable for the regulation of Bcl-2 expression in spermatocytes during spermatogenesis.

Another interesting finding of our study is the activation of non-canonical NFκB signaling in the CD147-deprived spermatocytes. Currently, the role of non-canonical NFκB signaling in regulating cell survival/apoptosis remains controversial. Several studies have indicated vital roles of its activation in the manifestation of malignant phenotype of cancer cells. Silence of NIK reduced cell growth and tumorigenicity of ovarian cancer cells [45]. Overexpressed NIK contributed to the tumorigenesis of adult T-cell leukemia and Hodgkin Reed-Sternberg cells [46]. On the other hand, non-canonical NFκB factors have been reported to promote apoptosis. The c-terminus of p100 contains a death domain, which is absent from all known tumor-derived mutants [37, 47]. The death domain mediates the recruitment of p100 into DISC to initiate the extrinsic apoptosis [37]. Moreover, non-canonical NFκB signaling has been shown to positively regulate the expression of death ligands FAS in pancreatic beta cells [36]. It has also been reported that p52 cooperates with p53 to regulate p53 target genes such as death receptor 5 in human osteosarcoma cell line U-2OS cells, suggesting a complex role for p52 as regulator for cell proliferation and apoptosis [48, 49]. However, the role of non-canonical NFκB signaling in germ cells is still largely unknown. The present results show that non-canonical NFκB signaling can be activated by depletion of CD147 in spermatocytes with increased levels of p100 and p52 and associated with the induction of extrinsic apoptosis. These results suggest that non-canonical NFκB signaling, when activated, promotes germ cell apoptosis, which is normally suppressed in the testis by the presence of CD147.

The current study has shown that depletion of CD147 down-regulates the expression of TRAF2, which alters both the canonical and the non-canonical NFκB signaling pathway. While the alteration on either arm of the NFκB signaling could result in apoptosis, whether the canonical, the non-canonical or both contribute to the CD147-regulated extrinsic apoptosis awaits further investigation. Nonetheless, we have shown that by resuming normal level of TRAF2 in CD147-depleted cells, the apoptotic activities can be reversed. These results suggest that CD147-regulated TRAF2 level acts upstream of NFκB signaling in regulating the apoptosis of spermatocytes.

In conclusion, the present findings have provided novel insights into the molecular mechanism underlying the regulatory role of CD147 in controlling germ cell survival/apoptosis, and thus spermatogenesis. The demonstrated role of CD147 in NFκB-dependent signaling governing germ cell survival/apoptosis may be relevant to cancer survival regulation since its expression is abnormally upregulated in various cancer [7]. Thus, the currently demonstrated CD147-regulated TRAF2 and its downstream signaling pathways in spermatogenesis may have broader implications in cancer biology and oncotargets.

## MATERIALS AND METHODS

### Animals

Eight-week old male severe combined immunodeficiency (SCID) mice were purchased from Laboratory Animal Services Centre of The Chinese University of Hong Kong (CUHK). All experiments were performed under the license from the government of the Hong Kong Special Administrative Region and were endorsed by the Animal Experimentation Ethics Committee of CUHK.

### *In vivo* seminiferous tubule injection

The male SCID mice seminiferous tubules injection was carried out as described, with slight modification [50]. Briefly, SCID mice were anesthetized and the testes were exteriorized through a midline abdominal incision. Mouse anti-CD147 antibody suspension (40 μg/mL, dissolved in 0.04% trypan blue with 10 μL total volume) was injected into the seminiferous tubules through the efferent duct. The testis filled about 70% of the surface area was considered to be successful injection [31]. As controls, an equal amount of normal mouse immunoglobulin G (IgG) was injected into the contralateral seminiferous tubules of the same mice. Nine days after injection, the total protein of testes was harvested in the RIPA lysis buffer (150 mM NaCl, 50 mM Tris-Cl, 1% NP-40, 0.5% deoxycholic acid, 0.1% SDS) plus protease inhibitor (Thermo Fisher Scientific) and stored at -80 ºC.

### Cell culture

Mouse spermatogonia cell line GC-1, mouse spermatocyte cell line GC-2 and HEK 293 cell line were purchased from the American Type Culture collection (ATCC) (ATCC number: CRL-2053, CRL-2196 and CRL-1573 respectively). They were grown in Dulbecco's modified Eagle's medium (DMEM) supplemented with 10% heat-inactivated fetal bovine serum and 1% penicillin–streptomycin and cultured in 5% CO_2_ incubator at 37 °C.

### Immunodepletion of CD147 in germ cells

Cells were seeded in 6-well plate at 5×10^4^ cells/well. After 24 h culture, the medium was replaced by fresh medium containing 10 μg/mL mouse anti-CD147, and normal mouse IgG at the same concentration was used as negative control. After 48 h antibody treatment, total cell protein was extracted with RIPA lysis buffer.

### CRISPR/Cas9 knockout of the mouse CD147 gene

Mouse CD147 gene (Accession: NM_001077184.1) contains 8 exons, and we designed the gRNA1 and gRNA2 (gRNA1/2) to knockout exon 2, 3 and 4, resulting in the frameshift mutation in CD147 gene (Supplementary Figure S2) and completely knockout of CD147 in GC-2 cells. Firstly, GC-2 cells were transduced with lentivirus expressing Cas9 nuclease and stably transduced cells were selected with 4 μg/mL puromycin. Then, gRNA1/2 lentiviruses were transfected into GC-2-Cas9 stable cell line for knockout CD147. After 72 h, DNA was extracted from the transfected cells using DNA extraction kit (QIAGEN) according to the manufacturer's instructions and protein was extracted with RIPA buffer, respectively. The Cas9 lentivirus and gRNA1/2 lentiviruses were purchased from GenePharma Company.

### Plasmid transfection and virus transduction

For transfecting TRAF2 overexpressing plasmid into HEK293, cells were seeded at density of 6 × 10^6^ cells per 100 mm dish. Transfection was performed using 36 μL Lipofectamine 2000 (Invitrogen) and 18 μg of pEBB-3XMyc-TRAF2 plasmid (Addgene plasmid # 44107) [51], according to the manufacturer's instructions. For transducing TRAF2-overexpressing lentivirus into GC-2, overexpression of TRAF2 in GC-2, cells were seeded into 24-well plate at 1 × 10^4^ cells/well with 200 μL DMEM plus 10% FBS. After 1 day, the medium was replaced with fresh medium and cells were transduced with 5 × 10^6^ TU lentivirues plus 0.1 % polybrene. Stable transduced cells were selected with 4 μg/mL puromycin after 48 h transfection.

### RNA extraction, reverse transcription, DNA extraction and PCR

Total RNA was extracted using Trizol reagent (Invitrogen), according to the manufacturer's instructions. cDNA was synthesized from 1 μg total RNA with iScript cDNA synthesis kit (Bio-Rad) according to the manufacturer's instructions. DNA was extracted with DNA extraction kit (Qiagen) according to the manufacturer's instructions. PCR assay was performed using with CD147 knockout primers (forward, 5’- TCG CTC TGA CTG TCT ACT GCA AA - 3’; reverse, 5’- AGA GTC TGT CCT ACA CTG TCC CA - 3’). The reaction mixture contained 2.5 μM of each primer, 25 mM MgCl_2_, 10 mM dNTP, 1-10 ng DNA template, 1×colorful PCR buffer and 0.2 μL Taq polymerase (TaKaRa). After 95°C denaturation for 5 minutes, the reactions were cycled 30 times with 10 seconds at 95°C, 30 seconds at 55°C, and 30 seconds at 72°C. At last, they were maintained at 72°C for 10 minutes.

### MTS cell viability assay

MTS cell viability assay was carried out as described in [38], cells were seeded into 96-well plate at 1 × 10^3^ cells/well with or without anti-CD147. OD_490 nm_ values were determined at indicated time points.

### Co-immunoprecipitation

Co-immunoprecipitation (Co-IP) was conducted according to the manufacturer's instructions (GE Healthcare). Briefly, 5 μg antibody was added in binding buffer (50 mM Tris, 150 mM NaCl, pH7.5), and 20 μL magnetic bead slurry medium was equilibrated with 500 μL binding buffer. Immediately after equilibration, the antibody solution was added in binding buffer with magnetic bead, incubated for 30 min. After antibody binding, the beads were washed with 500 μL binding buffer. Cells were lysed with 1 mL IP lysis buffer (50 mM HEPES, 420 mM KCl, 0.1% NP-40, 1 mM EDTA) on 2^nd^ day after transfection, added into the antibody-bond beads and incubated overnight at 4°C. Then, the beads were washed 3 times with wash buffer (50 mM Tris, 150 mM NaCl, pH7.5). At last, the proteins bound to the beads were eluted by 20 μL 1×SDS loading buffer (Thermo Fisher Scientific).

### Western blotting

Animal tissues and cells were lysed with RIPA buffer. Western blotting analysis was performed as described in our previous study [38]. Total cell lysates (30 μg per lane) were subjected to SDS-PAGE electrophoresis and were transferred onto nitrocellulose membranes. The transferred membrane was blocked with 5% fat-free milk in TBST (50 mM Tris-HCl, 150 mM NaCl, and 0.05% Tween 20, pH 8.0) for 1 h followed by overnight incubated with the primary antibodies (Supplementary Table S1) at 4°C in TBST with 2% fat-free milk. The membrane was incubated for 1 hour with appropriate peroxidase-conjugated secondary antibodies after 3 times TBST wash. The membrane was detected by enhanced chemiluminescence (GE Healthcare Biosciences) after 3 times TBST wash. The developed films were quantified by densitometry with Alpha Imager HP. Equal areas surrounding the bands were measured for bands of interest, background subtracted and integrated density value was calculated, and then normalized to the loading control (β-tubulin/β-actin).

### Statistical analysis

The *t*-test analysis was used for comparison between two measurements. Statistical significance for evaluation of three measurements was determined by one-way analysis of variance. All statistical analyses were performed using Prism 6.0 (GraphPad Prism). Differences were considered to be statistically significant at *P*< 0.05.

### Funded by

- National 973 Projects. Grant Numbers: 2013CB967401, 2013CB967404.
- National Science Foundation of China. Grant Numbers: 81671432, 31071019, 31140034.
- Science and Technology Planning Project of Guangdong Province: 2016A020218005.
- Fund for high level medical discipline construction of Shenzhen: 2016031638.
- Focused Investment Scheme of the Chinese University of Hong Kong.
- Hong Kong University Grants Committee. Grant Number: GRF/CUHK/466413.
- Chaoqun Wang was supported by the Hong Kong PhD Fellowship Scheme (HKPFS) issued by RGC of Hong Kong.

## SUPPLEMENTARY MATERIALS FIGURES AND TABLE


