# Supplementary material for: CD147 regulates extrinsic apoptosis in spermatocytes by modulating NFκB signaling pathways
Source: Oncotarget. 2016 Nov 25;8(2):3132–43. doi: 10.18632/oncotarget.13624 (PMC5356870; doi:10.18632/oncotarget.13624)
Supplement: Supplementary file 1 [file oncotarget-08-3132-s001.pdf]

CD147 regulates extrinsic apoptosis in spermatocytes by modulating NFκB signaling pathways

SUPPLEMENTARY FIGURES AND TABLE

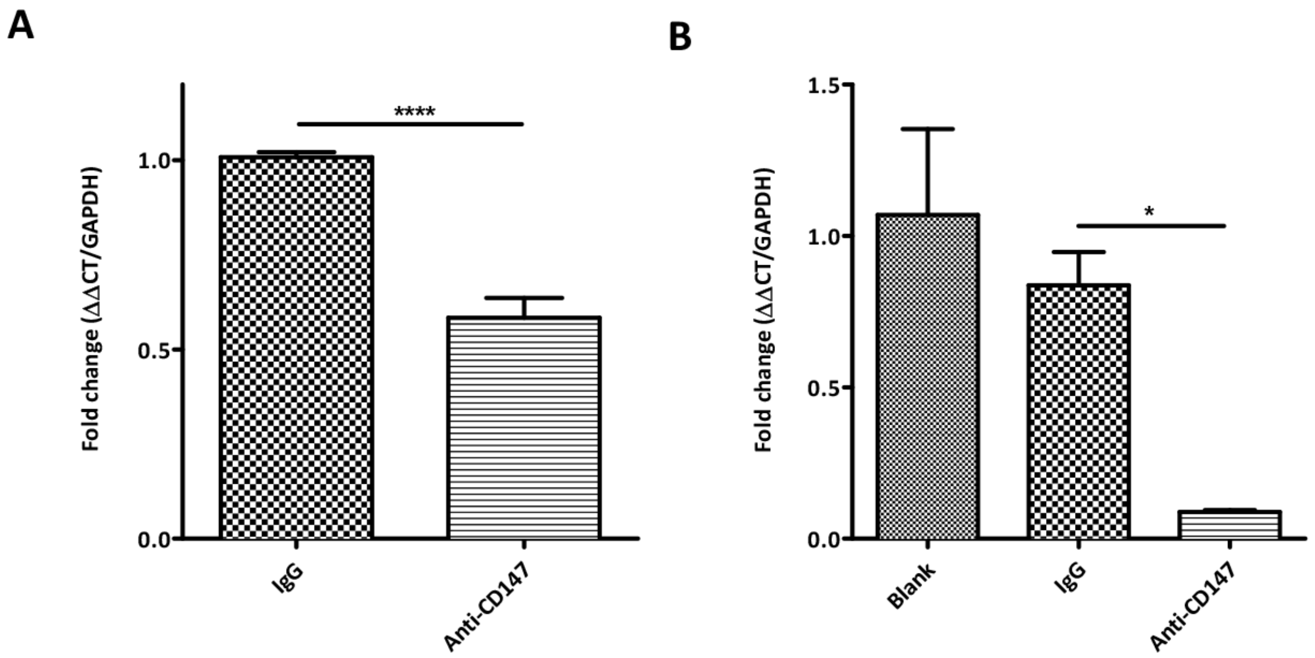

Supplementary Figure S1: Real-time PCR analysis of TRAF2 in CD147-immunodepleted testis of SCID mouse (A) and anti-CD147 treated GC-2 cells (B). The experiments were repeated 3 times. Values represent the mean±SEM. *, $p<0.05$, ***, $p<0.001$.

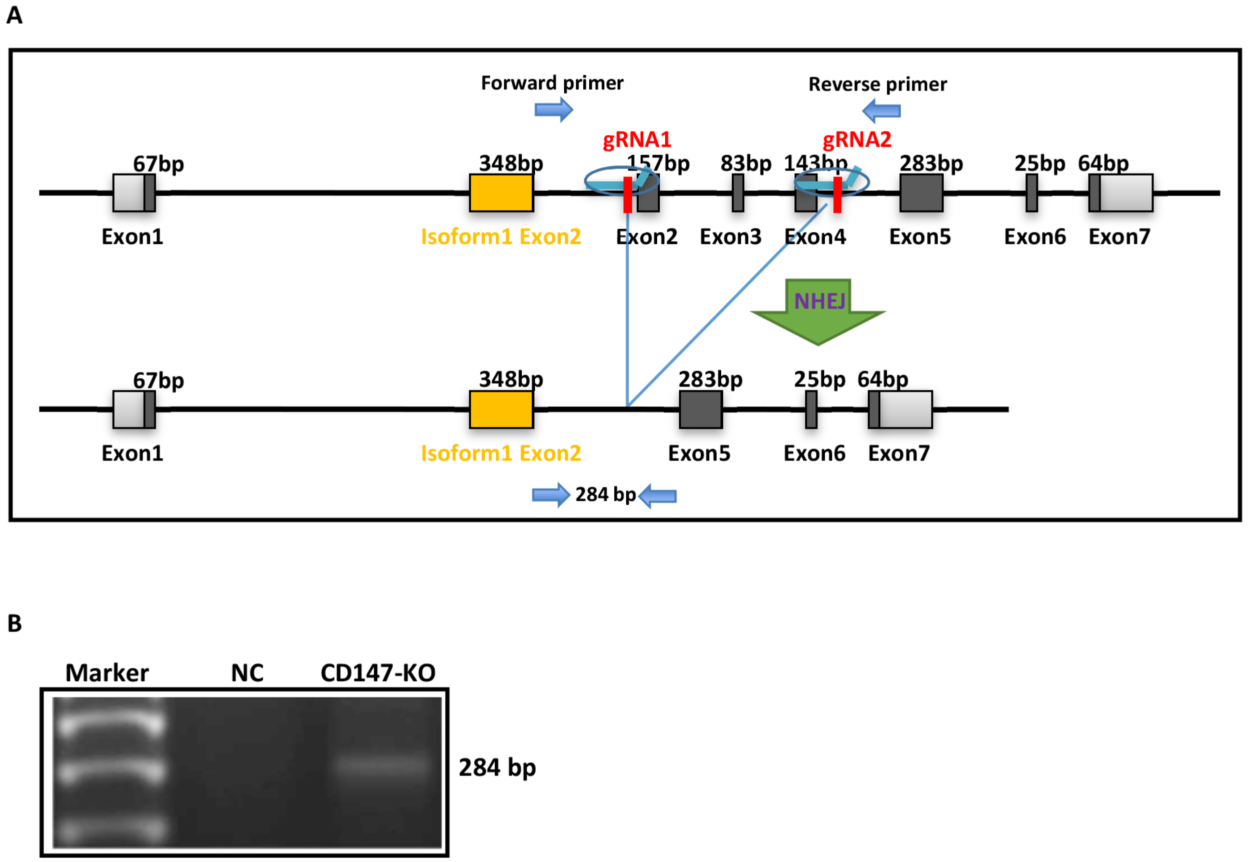

Supplementary Figure S2: CRISPR/Cas9-mediated knockout of CD147. A. Schematic diagram showing the sgRNA design targeting exon 2 – 4 of the mouse *CD147* gene. **B.** Genomic PCR showing the presence of recombined allele (284 bp after removal of exons 2 – 4) of *CD147* gene in sgRNA transfected cells (CD147-KO) but not the vector control transfected cells (NC).

Supplementary Table S1: Antibodies used in this study

Name	E$^{\Delta}$	Vendor	Catalog No.	Dilution
Anti-CD147 (M6/1)	FB	SCB*	sc-51591	Testis: 40 μg/mL, 10 μL; Cell: 10 μg/mL
Anti-CD147 (G19)	WB	SCB	sc-9757	1:500
Anti-pro-caspase-3/cleaved caspase-3	WB	CST	9662	1:1000
Anti-cleaved caspase-3	WB	CST	9664	1:1000
Anti-pro-caspase-8	WB	CST	4927	1:1000
Anti-cleaved caspase-8	WB	CST	9429	1:1000
Anti-PARP	WB	SCB	sc-25780	1:500
Anti-cleaved PARP	WB	CST	9544	1:1000
Anti-TRAF2	WB	CST	4712	1:1000
Anti-c-Myc	IP	Provided by Dr. YU Siu Bun	25 μg	
Anti-c-Myc	WB	CST	2276	1:1000
Anti-p105/p50	WB	CST	12540	1:1000
Anti-p65	WB	SCB	sc-109	1:500
Anti-NIK	WB	CST	4994	1:1000
Anti-p100/p52	WB	CST	4882	1:1000
Anti-Rel B	WB	CST	4922	1:1000
Anti-β-tubulin	WB	SCB	sc-9104	1:1000
Anti-β-actin	WB	Sigma	A1978	1:2000

$^{\Delta}$Experiment, †Functional blocking, ‡Western blot, *Santa Cruz Biotechnology, #Cell Signaling Technology.
